# Assessment of self-efficacy for caregiving in oncology: Italian validation of the caregiver inventory (CGI-I)

**DOI:** 10.1186/s12904-021-00849-5

**Published:** 2021-10-21

**Authors:** S. Serpentini, B. Guandalini, G. Tosin, L. Ronconi, G. Cristaldi, R. Amatulli, G. Deledda, S. Riccardi, S. Sommacal, L. Iannopollo, V. Calvo, T. V. Merluzzi

**Affiliations:** 1 Veneto Institute of Oncology IOV – IRCCS, Padua, Italy; 2 Department of Philosophy, Sociology, Pedagogy and Applied Psychology (FISPPA) University of Padua, Padua, Italy; 3 Clinical Psychology Unit, IRCCS Sacro Cuore Don Calabria Hospital, Negrar di Valpolicella, Verona, Italy; 4 Fondazione Policlinico Universitario A. Gemelli, Università Cattolica del Sacro Cuore, Rome, Italy; 5 Department of Psychology, University of Notre Dame, Notre Dame, IN 46556 USA

**Keywords:** Self-efficacy, Caregiving, Cancer, Validation, Caregiver inventory, Depression, Anxiety, Stress

## Abstract

**Background:**

The Caregiver Inventory (CGI), a measure of self-efficacy for caregiving that takes into account aspects of caregiving that are neglected by current measures of caregiving, was translated into Italian and validated.

**Methods:**

Ninety-one caregivers from a variety of locations in Italy completed the CGI-Italian (CGI-I) as well as the Hospital Anxiety and Depression Scale (HADS) and the Family Strain Questionnaire - Short Form (FSQ-SF).

**Results:**

A confirmatory factor analysis based on the original CGI factor structure resulted in an adequate fit of the CGI-I using standard fit indices. Thus, the original factor structure was validated in the CGI-I: Managing Medical Information (α = 0.87), Caring for Care Recipient (α = 0.68), Caring for Oneself (α = 0.78), and Managing Difficult Interactions/Emotions (α = 0.55). The CGI-I total score was inversely related to anxiety (HADS, r = − 0.35, p = <.05), and depression (HADS, r = − 0.45, p = <.05). In addition, the CGI-I was inversely related to caregiver stress (FSQ-SF, r = − 0.39, p = <.05). Care of Oneself and Managing Difficult Interactions/Emotions emerged as the strongest and most robust negative relationships with anxiety, depression, and caregiver stress, which replicated, with similar constructs, findings from the original CGI.

**Conclusions:**

The results of this study established the CGI-I as a reliable and valid measure of self-efficacy for caregiving. This study also confirms the importance of self-care and managing difficult communication in the process of successfully navigating the demands of caregiving and in constructing interventions for caregivers who need support.

**Supplementary Information:**

The online version contains supplementary material available at 10.1186/s12904-021-00849-5.

## Introduction

Caregiver support plays an increasing role in the lives of those with a diagnosis of cancer because of the rising incidence of cancer world-wide and longer survival times of those with advanced and terminal disease. This combination of increased incidence and longevity has resulted in the need for greater assistance from informal caregivers [1]. According to current data, approximately 3 million people serve as informal caregivers to cancer patients each year and devote an average of 32 hours per week on caregiving tasks, which include medical and nursing tasks [2] especially in the case of patients with advanced cancer. Those data also show that women generally assume the caregiver role most frequently and in a particular way that is different from men. Whereas men are more interested in the organizational and practical aspects of caregiving, women do more direct care of the patient and for that reason there is a greater engagement with the burden of care and its consequences [3]. Even in countries where there is support for caregiving, such as Italy, there are significant personal and financial burdens involved in caregiving [4] and interventions to relieve those burdens [5]. Considering that the cost of long-term care is increasing and the population that needs caregiving is growing, the numbers of family members who provide caregiving is increasing substantially [1, 6]. This situation has resulted in a focus on a closer analysis of the demands and consequences of caregiving [7], dimensions of caregiving [8], and models of caregiving that might be based in agency rather than burden [8].

Caregivers are typically people in the patient’s life who respond to their health, emotional, financial, and spiritual needs through all stages of the disease. However, they are generally not necessarily trained for many of the tasks they may be required to perform and may also not be prepared for the emotional consequences, including *primary stress* related to direct caregiving and *secondary stress*, which may affect the quality of the caregiver’s physical, psychological, social and financial quality of life [4]. Thus, caring for a loved one is generally associated with significant *caregiver burden*, which is defined as a subjective experience of stress that occurs when an imbalance exists between caregiving demands and caregiver resources to cope with those demands [9]. The burden of caregiving for someone with cancer is somewhat different than other diseases because cancer symptoms vary from person to person and may involve more complexity because cancer may affect many bodily functions especially if the cancer is metastasizing. Therefore, caregivers have to monitor the patient’s health frequently and also must engage in a variety of coping skills to deal with the emotional aspects of their own lives and those for whom they are providing care [10]. For this reason, cancer caregiving may significantly impact the psychological and physical health of family members [11].

Along these lines, research indicates that those who take care of cancer patients have significantly more anxiety and depression than the normal population and other caregivers [9, 12]. In addition, about two thirds of caregivers (62%) are in what has been termed a “high burden” situation with high levels of psychological distress that may not remit over time. According to the findings regarding physical burden, typical physical symptoms found in caregivers are insomnia, immunosuppression, cognitive decline, fatigue, chronic pain, changes in appetite as well as typical psychological symptoms that include anxiety, depression, loss of hope and negative emotions. These factors often impact other domains of the caregiver’s life in terms of reducing work productivity, increasing isolation, intensifying difficulties in accomplishing daily routines and aggravating problems with seeking medical information and communicating with medical staff [2, 7, 11]. Thus, caregivers may experience problems in the relationship between them and the person for whom they are providing care, in addition to economic problems, recurrence of previous psychological disorders and declines in health status [13]. However, there is emerging evidence that there may be other aspects of caregiving (e.g., self-care, dealing with negative emotions from the person for whom they are providing care) rather than actual caregiving tasks that are the causes of these emotional and physical problems that accompany caregiving. Moreover, these difficulties may represent a loss of agency or self-efficacy for managing aspects of caregiving other than direct care [8].

In their critique of the caregiving literature, Merluzzi, Philip, Vachon and Heitzmann [8] noted a number of limitations in the models and measurement of caregiving that typically only reflected caregiver stress and burden. Among other things, Merluzzi et al. [8] noted that because burden was the focus of caregiving research, there was a lack of acknowledgement of the importance of the interpersonal relationship between the caregiver and the person receiving care and the communication between them. Thus, a focus on the relationship in the context of caregiving changes the perspective from merely attending to caregiver tasks and burden to including the quality of the relationship, which is consistent with a broader definition of the caregiver role. In addition. Merluzzi et al. [8] noted that in the extant literature there was a neglect of positive aspects of caregiving. In spite of the difficulties that caregiving entails, caregivers do perceive value in their efforts and acknowledge that there is a positive side to their caregiving role. Finally, Merluzzi et al. drew attention to the fact that in spite of a growing focus on caregiver self-care, the assessment of caregiving did not include self-care. That is, the research had established that when caregivers pay more attention to themselves and their own health, there is a consequent decrease of stress and burnout associated with their caregiver role [14]. However, in assessing caregiving, self-care was notable absent in measures in favor of focusing solely on stress and burden.

Based on those critiques of the current state of caregiving measurement, Merluzzi et al. developed the Caregiver Inventory (CGI), which was grounded in self-efficacy theory [15, 16] and focused not only on caregiver tasks but also on the relationship between the caregiver and the person being cared for, the positive aspects of caregiving and caregiver self-care. According to self-efficacy theory, caregivers assess the caregiving situation in terms of the demands of completing caregiving behaviors in relation to their expectancy that they can successfully engage in those tasks and optimize valued outcomes. Thus, caregivers who have high expectations of their ability to provide assistance with bathing and cleanliness may produce desired outcomes such as the comfort of the person being cared for, satisfaction with one’s role as a caregiver, the experience of positive emotions, or not “being burned out” compared to those with low self-efficacy expectations. Thus, self-efficacy is tantamount to a sense of agency [15], and with respect to caregiving, self-efficacy refers to the expectancies that people have not only about their ability to competently support the patient but also their ability to execute of coping strategies, especially in relation to their tenacity in attempting overcoming setbacks and the duration of their commitment to achieve their goals. Thus, the role of self-efficacy in cancer management is crucial because expectations of caregiving efficacy are inversely related to psychological distress, physical functioning, stress, burden and burnout and positively related to benefits for the patient [8, 17].

Given the novel and multidimensional nature of caregiving that is reflected in the Caregiver Inventory [8] and the quality of that measure, the purpose of the current study was to conduct a validation of the Italian version of the Caregiver Inventory (CGI-I), a non-disease-specific measure of self-efficacy for caregiving. The CGI-I is expected to be a valuable measure for research and clinical work because of the innovation of the original CGI with respect to its relationship orientation, the focus given to caregivers’ own self-care and the attention to the positive aspects of caregiving. Thus, the CGI goes beyond the only other self-report measure of self-efficacy [18], the Caregiver Self-Efficacy Scale, which does not include important relational and communicative factors, does not examine caregiver self-care and does not attend to any positive aspects of caregiving. Indeed, the ability to identify the positive aspects of caregiving such as discovering personal strength and personal growth in adversity has been linked with coping and finding meaning in caregiving [19]. Moreover, as noted earlier, in contrast to the CGI, other measures of caregiving focus more narrowly on stress and burden to the exclusion of aspects that provide a more complete understanding of caregiving [18, 20].

We hypothesized that the factor structure of the original CGI would be confirmed in the CGI-I on a sample of Italian caregivers. In addition, we hypothesized that the CGI-I would be internally consistent and concurrently valid in relation to other relevant constructs such as anxiety, depression, and caregiver stress and strain.

## Methods

### Participants

Caregivers were recruited according to the following inclusion criteria: at least 18 years old and providing informal caregiving for a person with a diagnosis of cancer. Exclusion criteria were the following: insufficient language skills to complete the questionnaires, deficits in functional capacity or clinical conditions that impired completion of the questionnaires autonomously (e.g., major psychiatric disorders, intellectual disabilities, or cognitive deterioration related to people, place or time).

The participants who met the inclusion criteria and consented to participate consisted of 91 caregivers of cancer patients. The age range of the caregivers was from 19 to 80 years and the mean age was 49.5. There was a higher prevalence of women (71.43%) and those who were married (71.43%). The majority of participants had a middle school diploma (36.46%) or a high school diploma (38.46%) and many were employed (46.15%). Concerning the family relationship with the patient, 50.55% were spouses, 21.98% children and 14.29% parents. There was a distribution in terms of length of caring for the patient ranging from “less than two months” to “more than 5 years.” Furthermore. 67.03% of caregivers lived in the same house as the patients and only 27.47% had a previous experience as a caregiver. The majority of the patients were in the therapeutic phase (54.95%) of cancer care. More detailed information about the caregiver sample is contained in Table 1.

**Table 1:** Demographic and caregiving information about the participants

**Sex**	***n (%)***
Males	26 (28.57%)
Famales	65 (71.43%)
**Partner Status**	***n*** **(%)**
Unmarried	14 (15.38%)
Married	65 (71.43%)
Cohabitant	6 (6.59%)
Widower	0
Separated/ Divorced	6 (6.59%)
**Education**	***n*** **(%)**
Primary school diploma	8 (8.79%)
Lower middle school diploma	33 (36.46%)
High school diploma	35 (38. 46%)
University Degree	6 (6.59%)
Others	9 (9.89%)
**Current Employment**	***n*** **(%)**
Employed	42 (46.15%)
Retired	24 (26.37%)
Homemakers	19 (20.88%)
Others	6 (6.59%)
**Relationship with the Patient**	***n*** **(%)**
Spouse/ Partner	46 (50.55%)
Son	20 (21.98%)
Grandson	2 (2.20%)
Parent	13 (14.29%)
Brother/ Sister	9 (9.89%)
Others	1 (1.10%)
**Length of Time in Caregiving for the Patient**	***n*** **(%)**
Less than two months	15 (16.48%)
Less than six months	15 (16.48%)
Less than one year	16 (17.58%)
Less than two years	14 (15.38%)
Less than five years	19 (20.88%)
More than five years	12 (13.19%)
**Living with the Patient**	***n*** **(%)**
Yes	61 (67.03%)
No	30 (32.97%)
**Previous Experience as a Caregiver of a Person with Cancer**	***n*** **(%)**
Yes	25 (27.47%)
No	66 (72.53%)
**Stage of the Patient’s Disease**	***n*** **(%)**
Diagnostic phase	1 (1.10%)
Therapeutic phase	50 (54.95%)
In remission	6 (6.59%)
Advanced stage	14 (15.38%)
Terminal phase	6 (6.59%)

Based on the information that the participants provided in the Family Strain Questionnaire-Short Form (FSQ-SF), descriptive data about the care-recipients were compiled. The average age of patients was 63 years old with a range from 38 to 88 years; 39 patients were male (42.86%), 46 were female (50.55%) and for six care recipients gender data was missing. The participants also indicated the type of cancer: breast, ovary and lung were the most frequent types of cancer but overall there was considerable variability in diagnosis.

### Procedure

In order to avoid limiting the validation of the CGI-I to a particular Italian geographical area, Veneto Oncology Institute (IOV) created a partnership with the “Sacro Cuore-Don Calabria” Hospital in Verona and the University Hospital “A. Gemelli” in Rome. The survey for the study was presented in two phases: first, the patients, who were receiving treatment at the clinics affiliated with the hospitals, were approached by a psychologist and, with the assent of the patient, the caregivers were contacted. The caregivers who volunteered to participate in the study, did so in the clinic setting in which they signed a consent form that was written according to the procedures established by current laws and regulations with regard to confidentiality and ethical standards. The caregivers were given a survey which took about 20 minutes to complete. A number code was used on the survey instead of the name of participants to preserve their privacy. Thus, no identifying information appeared on any of the study materials.

### Instruments

The researchers at each site administered a folder containing: the informed consent form that also described study’s objectives, a personal data sheet to obtain socio-demographic information and three self-report questionnaires: the Italian version of the Caregiver Inventory [8] that was the focus of this validation study as well as the Hospital Anxiety and Depression Scale [21, 22] and the Family Strain Questionnaire-Short Form [23] that were already validated and used in research with Italian participants.

#### Personal data sheet

The personal data sheet form was composed of items about socio-demographic characteristics, including the caregiver’s age, sex, marital status, level of education, occupation, partnership status, family relations, length of the caregiving period and prior caregiving experience.

#### Caregiver inventory (CGI)

The CGI [8] was designed to assess caregiving self-efficacy expectations of caregivers, who were providing informal caregiving for people with illnesses. The CGI, which had a Cronbach’s alpha of 0.91, is composed of 21 items. Participants responded to items using a 9-point Likert-type scale ranging from 1 (not at all confident) to 9 (totally confident). A total score consisted of the sum of the responses to all 21 items. In addition, based on a factor analysis of the original CGI, four subscales were derived and scores on the subscales are determined by summing the items in each factor: Factor 1:Managing Medical Information (α = 0.64), items 1, 14 and 19; Factor 2: Caring for the Care Recipient (α = 0.78), items 2, 6, 7, 8, 13, 18 and 21; Factor 3: Caring for Oneself (α = 0.88), items 3, 9, 11, 15 and 16; Factor 4: Managing Difficult Interactions/Emotions (α = 0.76), items 4, 5, 10, 12, 17 and 20. The CGI was inversely correlated with stress (Perceived Stress Scale, r = − 0.54. *p* = 0.001) [24] and burden (Caregiver Burden Inventory, r = − 0.37. p = 0.001) [25]. Furthermore, Factor 3 (Caring for Oneself) and Factor 4 (Managing Difficult Interactions/Emotions) had the strongest negative correlations with stress and burden measures.

##### Translation of the CGI

The translation of the CGI was performed in accordance with the EORTC guidelines [26], which began with obtaining permission from the original authors [8] to translate the CGI into Italian. The initial translation from English was accomplished by two Italian native speakers with excellent English skills. The two translated versions were reviewed by a third person to resolve any differences. This preliminary Italian translation was reviewed by a native English speaker, who was also fluent in Italian and who back-translated the Italian version into English. That same person compared the back-translated version with the original English version and resolved differences in wording. Taking into account the back-translated English version, the original version, and the Italian versions, the Italian and English translators collaborated on a further revision that resolved differences and provided a consensus translation of the CGI into Italian (CGI-I). A pilot phase was subsequently conducted, which included: 1. An administration of the CGI-I to five caregivers who were representative of the sample included in this study; 2. a thorough interview to probe the caregivers’ understanding of the items; 3. the collection and recording of any perceived difficulties with items; 4. an analysis of the CGI-I data to determine if there were any aberrant items. Based on the pilot phase, very minor modifications were made to establish the final version the CGI-I for validation in this study. The final version of the CGI-I was back-translated into English sent to the original authors, who evaluated and approved of that version.

#### Hospital anxiety and depression scale (HADS)

The HADS [22] was created to assess anxious (HADS-A) and depressive (HADS-D) symptoms within a hospital setting. As with the original version, the Italian version [21] includes 14 items that ask the respondent to report feelings experienced during the previous week on 4-point Likert-type scales (ranging from 0 to 3) that vary with the items but generally assess frequency. The scoring procedures consisted of summing the item ratings to compute scores for HADS-A and HADS-D as well as a global score (HADS-A + HADS-D). Given that the mean HADS-A score for the caregivers in this study’s sample, 8.96, and the mean HADS-D score, 7.37, were above 7, the participants were, on the average, in a mild range on both anxiety and depression and in the mild distress (anxiety + depression) range with a total score of 16.33.

#### Family strain questionnaire- short form (FSQ-SF)

The FSQ-SF [23] is an assessment instrument aimed at examining the impact of chronic diseases of adult patients on family member’s and caregiver’s quality of life, characterized by stress and strain. The FSQ-SF includes a brief personal data sheet, which contains items intended to provide demographic data about the patient and caregiver. That is followed by 30 items that represent 5 factors: emotional overload (or burden), problems in social interactions, searching for information about the disease, satisfaction with family relationships and thoughts about death. The participants responded in a dichotomous format (yes-no) and scoring was achieved by summing the “yes” responses; if the total score is less than 20 and if there are any affirmative answers to items 24 through 30, the total score is increased by 1 point for each of those “yes” responses. Based on a mean score of 14.84 for the sample in this study, the participants, on the average, were in a category in which a psychiatric consultation would be highly recommended [23]. Thus, the sample can be described as experiencing significant stress and strain.

### Data analysis

Data analyses were performed using R® statistical software. A Confirmatory Factor Analysis (CFA) was computed in order to verify that the original [8] four-factor structure fit the current data*.* The original four factor structure in Merluzzi et al. [8] was based on an exploratory factor analysis of the CGI. In order to test that factor structure’s fit with the current Italian sample, the items that were identified as being associated with a particular factor because of high factor loadings on one factor and lower on others, were  used as the basis for testing whether the Italian version of the CGI replicated that factor structure. Thus, in the CFA, hypothesis testing proceeded by allowing those factor-consistent items to freely vary while constraining the remaining items to “0”. The quality of the model fit was tested using the following fit indices: chi-square over degrees of freedom ratio (χ^2^/ df); Comparative Fit Index-CFI [27]; Tucker Lewis index-TLI [28]; Standardized Mean Square Residual-SRMR [29]; and Root Mean Square Error of Approximation-RMSEA [30]. CFI and TLI values > 0.95, RMSEA <.06, SRMR < 0.08 and χ2/ df < 3 indicate acceptable fit [31, 32]. The fit statistics were based on the CFA model in which the factor-consistent items based on Merluzzi et al. [8] were allowed to vary and the remaining items were constrained to “0”.

Internal consistency of each scale was computed using Cronbach’s α and concurrent validity between CGI, HADS and FSQ-SQ was assessed with Pearson’s correlation coefficients. In order to explore risk factors, the relationship of demographic variables with the CGI factors was tested with select demographic variables as independent variables and the CGI-I factors as dependent variables in regression analyses. In addition, t-tests were computed with some dichotomous demographic variables to assess differences (e.g., sex, cohabitation) on the total score and subscales of the CGI-I.

## Results

### Confirmatory factor analysis

The fit indices from the Confirmatory Factor Analysis were acceptable, indicating that the factor structure (Table 2) replicated the original four-factor solution [8]. The loadings of the items on the respective factors were all significant, ranging from 0.62 to 0.79 for F1, from 0.58 to 0.78 for F2, from 0.56 to 0.84 for F3 and from 0.18 to 0.70 for F4 (Table 3). Finally, the covariances between factors were all significant, ranging between 0.49 and 0.94.

**Table 2:** Fit indices of the 4-factor model and 21 items of the Caregiver Inventory – Italian Version

***χ***^***2***^	df	***p***	***χ***^***2***^***/***df	CFI	TLI	RMSEA	SRMR
401.27	183	>.001	2.19	0.95	0.95	0.12	0.10

Note: χ^2^/ df = Chi-square over degrees of freedom ratio; CFI=Comparative Fit Index; TLI = Tucker Lewis index; SRMR= Standardized Mean Square Residual-SRMR; RMSEA = Root Mean Square Error of Approximation-RMSEA

**Table 3:** Item Factor Loadings for the CFA on the Caregiver Inventory – Italian Version

Factors→ Items	1 Managing Medical Information	2 Caring for the Care Recipient	3 Caring for Oneself	4 Managing Difficult Interactions and Emotions
CGI-1	0.62			
CGI-14	0.72			
CGI-19	0.79			
CGI-2		0.58		
CGI-6		0.61		
CGI-7		0.70		
CGI-8		0.60		
CGI-13		0.78		
CGI-18		0.83		
CGI-21		0.75		
CGI-3			0.56	
CGI-9			0.84	
CGI-11			0.71	
CGI-15			0.58	
CGI-16			0.70	
CGI-4				0.18
CGI-5				0.57
CGI-10				0.61
CGI-12				0.61
CGI-17				0.70
CGI-20				0.30

Note: The items contained in the Caregiver Inventory are listed in the online supplemental file 1

### Reliability analysis

The Cronbach’s alpha for the CGI-I total score was α = 0.87. For the single factors, the values were α = 0.68 for F1, α = 0.81 for F2, α = 0.78 for F3 and α = 0.55 for F4. The item-total score correlations for F1 ranged from 0.40 to 0.71, for F2 from 0.51 to 0.76, for F3 from 0.54 to 0.83 and for F4 from 0.21 to 0.61. Overall these data were satisfactory and indicated acceptable reliability for the CGI-I.

### Concurrent validity

All concurrent validity coefficients reflected an inverse relationship between the CGI-I and both the HADS and the FSQ-SF (Table 4). The strongest correlations obtained between CGI-I and both the HADS and FASQ questionnaires were with Factors 3 and 4 and also with the CGI-I total score, whereas the lowest correlations were those with Factors 1 and 2. This pattern of results is similar to the validation data for the original CGI, which showed that distress and burden were more highly inversely correlated with caring for oneself (Factor 3) and managing difficult interactions/emotions (Factor 4) compared to understanding medical information (Factor 1) and caring for the care recipient (Factor 2). Likewise, in the current study higher levels of depression, anxiety, and caregiver stress are more likely at lower levels of caregiver efficacy for caring for oneself and also managing difficult interactions/emotions with the person for whom care is being provided. These finding are similar to those found in the validation of the original CGI.

**Table 4:** Pearson Correlations Between the Caregiver Inventory – Italian Version, HADS and FSQ-SF

	CGI Total	CGI Factor 1	CGI Factor 2	CGI Factor 3	CGI Factor4
HADS Anxiety	− 0.35*	− 0.16	− 0.17	−0.46*	− 0.26*
HADS Depression	−0.45*	− 0.22*	− 0.16	− 0.56*	−0.42*
HADS Total	−0.43*	−0.20	− 0.18	−0.54*	− 0.36*
FSQ-SF Total	−0.39*	− 0.13	−0.23*	− 0.47*	−0.31*

Note: FSQ-SF = Family Strain Questionnaire- Short Form. Factor 1: Managing Medical Information; Factor 2: Care of the Care Recipient; Factor 3: Caring for Oneself; Factor 4: Managing Difficult Interactions and Emotions. * = *p* < .05

### Demographic variables and CGI-I

Exploratory analyses were conducted to examine the relationship between demographic data in relation to the CGI-I to identify potential risk factors. Regression analysis with “age” as a predictor indicated that it was related only to Factor 4 (b = .463; t = 2.076. *p* = 0.040 and Rsqr = 0.047). Thus, self-efficacy for managing difficult interactions/emotions increased somewhat with age. Another regression analysis with how much time caregivers spend looking after patients as the predictor was significantly negatively related to F4 (b = −.933; t = − 2.186. *p* = 0.031 and Rsqr = 0.051). Thus, perhaps older caregivers can manage difficult interactions and emotions involving caregiving better than younger caregivers, however the more time spent caregiving, the more difficult that becomes.

Taking the self-identified sex of the caregiver into account as an independent variable and CGI factors as dependent variables, results showed that there were some differences between men and women on the total CGI-I score (t(89) = 3.346. *p* = 0.0013), on Factor 3 (t(89) = 4.424. *p* = 001) and Factor 4 (t(89) = 2.712. *p* = 0.008). Males scored higher on the CGI total score (Men: M = 147.04; Women: M = 132.54) as well as on Factor 3 (Caring for Oneself; Men: M = 32.85.; Women: M = 24.97) and Factor 4 (Managing Difficult Interactions/Emotions; Men: M = 39.85 Women: M = 35.26) compared to women. Finally, those caregivers who cohabitated with the patient had higher (t(89) = 2.371. *p* = 0.020) Factor 4 (Managing Difficult Interactions/Emotion) scores (M = 37.85) compared to those who did not cohabitate (M = 33.97). Also, caregivers with prior experience in caregiving had higher (t(89) = 2.406. *p* = 0.18) scores (M = 22.88) on Factor 1 (Managing Medical Information) than those without prior caregiving experience (M = 20.63).

## Discussion

The main objective of this study was to validate the Caregiver Inventory in the Italian language. The confirmatory factor analysis fit indices were in an acceptable range, which confirmed that the four-dimensional structure of the original model established by Merluzzi et al. [8] fits the Italian sample in this study. Thus, the impetus for the development of the original measure, namely to expand the scope of the assessment of caregivers beyond the traditional perspective that focused stress and burden, also applies to the current Italian sample. Thus, the inclusion of self-care, the positive aspects of caregiving and the complexities of the relationship between the caregiver and the person being cared for, emerge as relevant in lives of caregivers in Italy. Moreover, viewing caregiving in terms of self-efficacy or agency with regard to caregiving is different than merely assessing caregiver tasks. The agentic perspective references the expectations of the caregiver to manage difficult situations, emphasizing competence even in very difficult circumstances versus mere performance of caregiving behaviors.

It is also important to note that the current study also replicates, with comparable constructs, the finding by Merluzzi et al. [8] that is it not the direct caring of the person that is solely associated with distress, stress, and burden, but also the efficacy to take care of oneself as a caregiver and to manage difficult and emotional interactions with the person receiving care. In essence, these results mean that performing the tasks of caregiving (e.g., assisting with activities of daily living, providing emotional support, providing support for medical treatments) may not be the sole cause of distress. Rather, contributing to distress in caregiving is the inability to engage in self-care (e.g., have a life apart from caregiving, seek support for oneself, take care of one’s own health) and the inability to manage emotional and sometimes difficult interactions with the person who is receiving care. These findings may help to focus supportive services for caregivers on respite and conflict management rather than on training on the traditional tasks of caregiving.

In essence, these results in the current validation of the CGI-I, like the original work on the development of the CGI, draw concerted attention both to the communicative elements between caregiver and patient and to the caregiver’s self-care behaviors. In particular, “managing difficult interactions and emotions” explores the interpersonal aspects of sharing and expressing feelings of suffering and anguish, which are often neglected even if they are fundamental for the patient’s psychological well-being. In fact, in a case of chronic or terminal illness, there may be what has been termed a *conspiracy of silence* [33], which is characterized by the presence of a relational understanding of conspiratorial silence between patient and family wherein the themes of illness and death become taboo. Moreover, this mutual detachment often produces further tension or suffering that cannot be named and processed openly. In addition, patients’ friends and family may contribute to the creation of this collaboration of silence because they do not recognize or are uncomfortable with the patients’ need to communicate their emotions. Consequently, patients seek other ways of sharing their concern through support groups and, more often recently, online social networks [34, 35]. Physical and psychological distress needs to be expressed and shared in order to avoid more serious conditions of loneliness, misunderstanding, and despair. The ability to engage in this interaction may help to mitigate caregiver anxiety, depression, and stress.

The results of the current study with regard to the CGI-I, and similarly, the original CGI, show that caregivers are often so busy with care tasks that they risk neglecting their interpersonal relationships and denying themselves the opportunity to take advantage of external social support. Caregivers may also consider their malaise less intense than the care-recipient’s and for this reason may not share their concerns. The CGI-I factor Caring for Oneself focuses on this critical and sometimes neglected practice of caregivers to subjugate their own well-being by focusing exclusively on the needs of the sick person perhaps to the detriment of their own health and well-being. This may be a consequence of both the actual lack of time to take care of themselves and the guilt for taking time for themselves instead of taking care of the person for whom they are caring [36]. The literature confirms that caregivers who neglect their own well-being have lower levels of quality of life and struggle to carry out care activities to the detriment of the patient [37]. Therefore, it is essential for the caregivers to take care of themselves and their interpersonal relationships; this may be possible only if the care burden does not fall entirely on the informal caregivers but can be provided to some extent by others either informally or through services designed to provide respite for caregivers. In summary, both in the original validation study and in the present study two aspects of caregiving, Managing Difficult Interactions/Emotions and Caring Oneself, correlate (inversely) highly with caregiver stress, burden, anxiety, and depression and should be the basis for interventions to elevate the quality of life of caregivers.

The results of the convergent validity analyses confirm prior findings of the inverse relationship between anxiety, depression, and burden scores and the CGI-I. Generally, the more caregivers feel efficacious in their role, the less they suffer from depressive and anxiety disorders and they experience less burden. In particular, as noted above, the third factor “Caring for Oneself” had the strongest inverse correlations with the HADS and FSQ-SF. In contrast, the factors Managing Medical Information and Care of the Care Recipient have, essentially little correlation with anxiety, depression, and caregiver stress. Thus, perceived competence in practical care activities may not be either a benefit or a detriment to the caregiver with respect to their emotional quality of life.

The Cronbach’s alpha values and the item to total score correlations indicate that the Caregiver Inventory (CGI) is a reliable measure. Furthermore, the results of this study with those of the original validation provide a concordance in the reliability values. Despite the lower alpha value for Factor 4, which is the only coefficient below the traditionally accepted threshold, its importance emerged in validity correlations, which indicated its significant and strong relationship with depression, anxiety, and caregiver stress. Factor 4 is also important given the complexity as well as the physical and emotional intimacy of the caregiving relationship, a profound and private relationship that includes themes of hope, loss and death. However, some of the items of the CGI deal with conflictual or problematic issues that may provoke varying degrees of reticence in terms of addressing them openly, especially in Italian culture. Evidence of varying degrees of reticence is probably also reflected in the lower factor loadings for items CGI-4 (*expressing negative feelings about the illness*) and CGI-20 (*dealing with criticism from others*), which may represent difficult issues in the Italian culture; however, even in the original factor analysis these items had the lowest factor loadings, perhaps indicating that in any culture these items represent very difficult issues in the context of caregiving provoking a wide range of responses.

Within this study, the relationships between CGI scores and socio-demographic variables were also explored in order to identify potential risk factors with regard to caregiving efficacy. The majority of caregivers in the sample were female, which corresponds to the literature showing that females more than males are frequently engaged in the caregiving role [3]. Despite this, males scored higher on the CGI-I total score and on Factors 3 and 4 indicating greater reported self-efficacy for caregiving. These findings highlight the tendency of men to adopt more agentic coping strategies as well as perhaps being less aware of their emotional response to the stress caused by caring for the patient [38].

The caregivers who participated in the study were mostly married to the cancer patient and were primary caregivers, who engaged in what becomes like a “second job” from which there is not much respite. On the one hand, living with the patient may exacerbate stress and impact emotional and physical well-being, but, on the other hand, it may help to sustain the quality of care and allow prompt action in case of emergency. Consistent with this, compared to caregivers who did not cohabitate with the patient, caregivers who cohabitated had higher scores on the Factor 4, confirming that physical proximity to the patient, in addition to helping with the management of practical burdens, may increase confidence to cope with emotional difficulties. In addition, previous caregiving experience is also related to CGI-I scores on Factor 1 (Management of Medical Information). Thus, previous experience in caregiving may have contributed to learning how to manage contact with medical-hospital environments, which provided enhanced perceived competence in this area of caregiving. Finally, older caregivers are more confident in managing difficult interactions and emotions with the person being cared for than younger caregivers, however as the time spent each day on caregiving increases, the more difficult it becomes to manage those difficult emotional interactions.

Self-efficacy for caregiving assumes that it is important for caregivers to have a sense of agency, however, in Italy caregivers are typically expected to not only invest in assisting the cancer patient, but also, to a great extent, be solely responsible for interfacing with with physicians and the health care staff as well as determining what should be shared with the patient. Under these cultural circumstances, the patient is less involved with the medical care team than in other cultures where the patient is involved in communicating directly with health professionals. Thus, the caregiver in Italy is likely heavily burdened by tasks of caregiving, manging the caregiving relationship, and dealing with information overload from the medical team, all of which is accompanied by poor self-care. In this cultural context, it would be very useful to provide support group services to caregivers in order to promote greater self-care and foster communication skills with health care providers that includes the patient’s involvment in order to reduce the caregiver’s burden.

### Limitations and future directions

The data in this study are cross-sectional, therefore caution must be exercised in interpreting causal connections in the results. For the sake of achieving a causal relationship between self-efficacy, depression and anxiety, these variables need to be investigated in longitudinal designs. Whereas, the sample size is modest, the replication of the original factor structure indicated that the same constructs apply to caregivers in Italy. Thus, confidence in the current findings is bolstered by the parallel results with the original CGI [8] However, being able to replicate the study with an enlarged sample could provide corroboration for the current findings [8]. The disproportionate number of females to males in the sample can be attributed to a greater tendency for women to take on caregiving roles in virtually every culture. However, the differences between men and women in caregiver self-efficacy in the current study should be explored more in future research because as more men engage in caregiving, interventions for enhancing caregiving may need to differ somewhat between men and women to optimize caregiving outcomes. Finally, it would be helpful in the clinical care context to develop a scoring system that might include cut scores for low levels of caregiving efficacy, perhaps based on the four factors, which would signal a need for support and training to increase the caregiver’s efficacy for caregiving.

Because the main objective of this work was to validate the CGI-I, only a few of the demographic variables were analyzed and reported. The effects of some of these variables in relation to the CGI-I have been presented, but each of them and others (e.g., disease-related variables, personality variables) deserve broader study, which could contribute the identification of risk factors for difficulties in caregivers. Finally, for the sake of external validity, it would be important to replicate this study on caregivers who are providing care to patients of diseases other than cancer.

## Conclusion

The CGI-I appears to be a structurally sound, reliable, and valid measure of self-efficacy for caregiving. In addition, the results reported in this study support the conclusion that improving self-care self-efficacy would lessen depression, anxiety, and caregiver stress. In particular, the results of this study and the original CGI study would suggest that interventions should focus attention on caregiver self-care and skills in managing conflict with the person for whom care is being provided.

## Supplementary Information

**Additional file 1.**

## Data Availability

The datasets used and/or analyzed during the current study are available from the first author on reasonable request.

## Abbreviations

CGI: Caregiver Inventory
CGI-I: Italian version of Caregiver Inventory
EORTC: European Organization for Research and Treatment of Cancer
HADS: Hospital Anxiety and Depression Scale
FSQ-SF: Family Strain Questionnaire- Short Form (FSQ-SF)

## References

[1] Zabora JR, Loscalzo MJ, Weber J (2003). Managing complications in cancer: identifying and responding to the patient’s perspective. Semin Oncol Nurs.

[2] Trevino KM, Prigerson HG, Maciejewski PK (2018). Advanced cancer caregiving as a risk for major depressive episodes and generalized anxiety disorder. Psycho-Oncology.

[3] Navaie-Waliser M, Spriggs A, Feldman PH (2002). Informal caregiving: differential experiences by gender. Med Care.

[4] Rossi PG, Beccaro M, Miccinesi G, Borgia P, Costantini M, Chini F, Baiocchi D, De Giacomi G, Grimaldi M, Montella M (2007). Dying of cancer in Italy: impact on family and caregiver. The Italian survey of dying of Cancer. J Epidemiol Community Health.

[5] Guida E, Barello S, Corsaro A, Galizi MC, Giuffrida F, Graffigna G, Damiani G (2019). An Italian pilot study of a psycho-social intervention to support family caregivers' engagement in taking care of patients with complex care needs: the engage-in-caring project. BMC Health Serv Res.

[6] Congressional Budget Office, United States of America. Long-Term Outlook for Health Care Spending. Congressional Publications. 2007. https://www.cbo.gov/sites/default/files/110th-congress-2007-2008/reports/11-13-lt-health.pdf.

[7] Robison J, Fortinsky R, Kleppinger A, Shugrue N, Porter M (2009). A broader view of family caregiving: effects of caregiving and caregiver conditions on depressive symptoms, health, work, and social isolation. J Gerontol.

[8] Merluzzi TV, Philip EJ, Vachon DO, Heitzmann CA (2011). Assessment of self-efficacy for caregiving: the critical role of self-care in caregiver stress and burden. Palliative Supportive Care.

[9] Hsu T, Loscalzo M, Ramani R, Forman S, Popplewell L, Clark K, Katheria V, Feng T, Strowbridge R, Rinehart R, Smith D, Matthews K, Dillehunt J, Hurria A (2014). Factors associated with high burden in caregivers of older adults with cancer. Cancer.

[10] Bolis T, Masneri S, Punzi S (2008). The caregiver in oncology: duties and needs. Giornale Italiano di Medicina del Lavoro ed Ergonomia.

[11] Vitaliano PP, Zhang J, Scanlan JM (2003). Is caregiving hazardous to One's physical health? A Meta-Analysis. Psychol Bull.

[12] Johansen S, Cvancarova M, Ruland C (2018). The effect of Cancer patients’ and their family caregivers’ physical and emotional symptoms on caregiver burden. Cancer Nurs.

[13] Geng H, Chuang D, Yang F, Yang Y, Liu W, Liu L, Tian H (2018). Prevalence and determinants of depression in caregivers of cancer patients: a systematic review and meta-analysis. Medicine.

[14] van den Heuvel E, de Witte LP, Schure LM, Sanderman R, Meyboom-de Jong B (2001). Risk factors for burn-out in caregivers of stroke patients, and possibilities for intervention. Clin Rehabil.

[15] Bandura A (1991). Social cognitive theory of self-regulation. Organ Behav Hum Decis Process.

[16] Bandura A. Self-efficacy: the exercise of control. New York: Freeman Press; 1997.

[17] Leung DYP, Chan HYL, Chiu PKC, Lo RSK, Lee LLY (2020). Source of social support and caregiving self-efficacy on caregiver burden and Patient’s quality of life: a path analysis on patients with palliative care needs and their caregivers. Int J Environ Res Public Health.

[18] Steffen AM, McKibbin C, Zeiss AM, Gallager-Thompson D, Bandura A (2002). The revised scale for caregiving self-efficacy: reliability and validity studies. The journals of gerontology. Ser B Psychol Sci Soc Sci.

[19] Wong PTP (2010). Meaning therapy: an integrative and positive existential psychotherapy. J Contemp Psychother.

[20] Vachon D. The Health Effects of Caregiving for Adults in Later Life. In: Whitman TL, Merluzzi TV, White RD, editors. Life-span Perspectives on Health and Illness, Erlbaum. Mahwah New Jersey; 1999. p. 239–60.

[21] Costantini M, Musso M, Viterbori P, Bonci F, Del Mastro L, Garrone O, Venturini M, Morasso G (1999). Detecting psychological distress in cancer patients: validity of the Italian version of the hospital anxiety and depression scale. Support Care Cancer.

[22] Zigmond AS, Snaith RP (1983). The hospital anxiety and depression scale. Acta Psychiatr Scand.

[23] Vidotto G, Ferrario SR, Bond TG, Zotti AM (2010). Family strain questionnaire – short form for nurses and general practitioners. J Clin Nurs.

[24] Cohen S, Kamarck T, Mermelstein R (1983). A global measure of perceived stress. J Health Soc Behav.

[25] Novak M, Guest C (1989). Application of a multidimensional caregiver burden inventory. The Gerontologist.

[26] Dewolf L, Koller M, Velikova G, Johnson C, Scott N, Bottomley A, Anonymous Brussels (2009). EORTC Quality of Life Group. 3rd ed.

[27] Bentler PM (1990). Comparative fit indexes in structural models. Psychol Bull.

[28] Tucker LR, Lewis C (1973). A reliability coefficient for maximum likelihood factor analysis. Psychometrika.

[29] Bentler PM (1985). Theory and implementation of EQS, a structural equations program.

[30] Steiger JH (2016). Notes on the Steiger-Lind (1980) handout. Struct Equ Model.

[31] Byrne RMJ (1989). Suppressing valid inferences with conditionals. Cognition.

[32] Hu L, Bentler PM (1999). Cutoff criteria for fit indexes in covariance structure analysis: conventional criteria versus new alternatives. Struct Equ Model.

[33] Testoni I (2016). Psicologia del lutto e del morire: dal lavoro clinico alla death education [the psychology of death and mourning: from clinical work to death education]. Psicoterapia e Scienze Umane.

[34] Stephen JE, Christie G, Flood K, Golant M, Rahn M, Rennie H, Speca M, Taylor-Brown J, Turner J (2011). Facilitating online support groups for cancer patients: the learning experience of psycho-oncology clinicians. Psycho-Oncology.

[35] Bol N, Rising C, Burke Garcia A, Rains S, Wright K (2017). Perceived stress in online prostate Cancer community participants: examining relationships with stigmatization, social support network preference, and social support seeking. J Health Commun.

[36] Nicholas Dionne-Odom J, Hooker S, Bekelman D, Ejem D, McGhan G, Kitko L, Strömberg A, Wells R, Astin M, Metin Z, Mancarella G, Pamboukian S, Evangelista L, Buck H, Bakitas M (2017). Family caregiving for persons with heart failure at the intersection of heart failure and palliative care: a state-of-the-science review. Heart Fail Rev.

[37] Neergaard, L. AP-NORC Poll: Many caregivers neglecting their own health. National Post (Online) 2018. https://www.longtermcarepoll.org/ap-norc-poll-many-caregivers-neglecting-their-own-health/.

[38] Lutzky SM, Knight BG (1994). Explaining gender differences in caregiver distress: the roles of emotional attentiveness and coping styles. Psychol Aging.

