# Supplementary material for: Assessment of self-efficacy for caregiving in oncology: Italian validation of the caregiver inventory (CGI-I)
Source: BMC Palliat Care. 2021 Oct 21;20:166. doi: 10.1186/s12904-021-00849-5 (PMC8529803; doi:10.1186/s12904-021-00849-5)
Supplement: Supplementary file 1 — Additional file 1. [file 12904_2021_849_MOESM1_ESM.docx]

**Appendix**

**Caregiver Inventory**

This questionnaire contains many things that a person might do when caring for someone. We are interested in your judgement of how confident you are that you can accomplish those things. Make sure your ratings accurately reflect your confidence whether or not you have done it in the past. So, your ratings reflect your confidence that you can do these things now (or in the near future).

Please read each numbered item. Then rate that item on how confident you are that you can accomplish that behavior. Circle a number on the scale. If you circle a “1” you would be stating that you are not at all confident that you can accomplish that behavior. If you circle a “9” you would be stating that you are totally confident that you can accomplish that behavior. Numbers in the middle of the scale indicate that you are moderately confident that you can accomplish that behavior.

Please rate all items. If you are not sure about an item, please rate it as best you can.

Not at all Moderately Totally

Confident Confident Confident

1 2 3 4 5 6 7 8 9

Not at all Moderately Totally

Confident Confident Confident

1 2 3 4 5 6 7 8 9

1 2 3 4 5 6 7 8 9

1. Coping with information overload
2. Listening and learning from the person as to how to care better for him or her
3. Letting go of things I can’t control
4. Expressing negative feelings about the illness
5. Maintaining hope
6. Being able to notice the “good moments” in caregiving when they occur
7. Allowing the person to have and express his or her own feelings
8. Assisting the person with activities such as feeding, washing, dressing, or toileting.
9. Continuing to take care of myself (for example: exercise, diet, sleep)
10. Talking openly and honestly with the person
11. Continuing to engage in personal activities that I like to do
12. Talking about death and dying
13. Providing emotional support to the person I’m caring for
14. Understanding medical information from doctors, nurses, or other sources.
15. Seeking support for myself
16. Dealing with feelings of helplessness
17. Dealing with the person expressing negative feelings toward you when they occur
18. Assisting and encouraging the person in following through with all treatments
19. Asking physicians and nurses questions
20. Dealing with criticism from others
21. Maintaining a close relationship with the person I’m caring for
